# RETRACTION: Long noncoding RNA CASC9 promotes the proliferation and metastasis of papillary thyroid cancer via sponging miR‐488‐3p

**DOI:** 10.1002/cam4.70302

**Published:** 2024-10-10

## Abstract

**Retraction:** Y. Chen, Y. Li, and H. Gao, “Long Noncoding RNA CASC9 Promotes the Proliferation and Metastasis of Papillary Thyroid Cancer Via Sponging miR‐488‐3p,” *Cancer Medicine* 9, no. 5 (2020): 1830–1841. https://doi.org/10.1002/cam4.2839.

The above article, published online on 13 January 2020, in Wiley Online Library (wileyonlinelibrary.com), has been retracted by agreement between the journal Editor‐in‐Chief, Stephen Tait, and John Wiley & Sons Ltd. A report submitted by a third party noted image duplications with other articles by different author groups. Specifically, the report found image duplications with other published articles in Figures 2D, 3C, 4B, 4C, 5C and 7B. The report also found image overlap between images in Figures 2C and 6H. The retraction has been agreed to because the evidence of image duplication across different articles, each of which describes different experimental conditions, fundamentally compromises the editors' confidence in the conclusions presented. The authors did not respond to our notice regarding the retraction.

**Retraction:** Y. Chen, Y. Li, and H. Gao, “Long Noncoding RNA CASC9 Promotes the Proliferation and Metastasis of Papillary Thyroid Cancer Via Sponging miR‐488‐3p,” *Cancer Medicine* 9, no. 5 (2020): 1830–1841. https://doi.org/10.1002/cam4.2839.

The above article, published online on 13 January 2020, in Wiley Online Library (wileyonlinelibrary.com), has been retracted by agreement between the journal Editor‐in‐Chief, Stephen Tait, and John Wiley & Sons Ltd. A report submitted by a third party noted image duplications with other articles by different author groups. Specifically, the report found image duplications with other published articles in Figures 2D, 3C, 4B, 4C, 5C and 7B. The report also found image overlap between images in Figures 2C and 6H. The retraction has been agreed to because the evidence of image duplication across different articles, each of which describes different experimental conditions, fundamentally compromises the editors' confidence in the conclusions presented. The authors did not respond to our notice regarding the retraction.

